# Comparing Microspheres with Different Internal Phase of Polyelectrolyte as Local Drug Delivery System for Bone Tuberculosis Therapy

**DOI:** 10.1155/2014/297808

**Published:** 2014-02-23

**Authors:** Gang Wu, Long Chen, Hong Li, Chun-Ling Deng, Xiao-Feng Chen

**Affiliations:** ^1^School of Materials Science and Engineering, South China University of Technology, Guangzhou 510640, China; ^2^National Engineering Center for Tissue Restoration and Reconstruction, Guangzhou 510006, China; ^3^Department of Materials Science and Engineering, Jinan University, Guangzhou 510632, China

## Abstract

We use hydrophobic poly(lactic-co-glycolic) acid (PLGA) to encapsulate hydrophilic ofloxacin to form drug loading microspheres. Hyaluronic acid (HA) and polylysine (Pls) were used as internal phase additives to see their influences on the drug loading and releasing. Double emulsion (water-in-oil-in-water) solvent extraction/evaporation method was used for the purpose. Particle size analysis display that the polyelectrolytes have low impact on the microsphere average size and distribution. Scanning electron microscope (SEM) pictures show the wrinkled surface resulted by the internal microcavity of the microspheres. Microspheres with HA inside have higher drug loading amounts than microspheres with Pls inside. The loading drug amounts of the microspheres increase with the HA amounts inside, while decreasing with the Pls amounts inside. All the polyelectrolytes adding groups have burst release observed in experiments. The microspheres with Pls internal phase have faster release rate than the HA groups. Among the same polyelectrolyte internal phase groups, the release rate increases with the amounts increasing when Pls is inside, while it decreases with the amounts increasing when HA is inside.

## 1. Introduction

Tuberculosis (TB) is a potentially fatal contagious disease which mainly affects pulmonary system. General method for the disease treating is 9 to 12 months of multidrug chemotherapy. In all diagnosed with this disease, 10 to 20 percent bone infection is reported [1, 2]. In complicated bone infection cases, infection foci debridement and internal fixation were chosen by doctors [3]. Apart from the surgery, antitubercular drug therapy was still indispensable for a time period [4, 5].

Filling the cavity after the debridement using autograft/allograft and implanted materials would remodel the defected bone and restore the function. The material implanted in the cavity after debridement serves as an “in vivo bioreactor” wherein the engineering of the neotissue is achieved by invocation of a healing response within the bioreactor space. Providing the implanted scaffold with the *in situ* drug release function is a promising drug administration mode to therapy the patients infected by bone tuberculosis. It offers a more effective method for specific site delivery, drug dosage optimizer, and release duration simultaneously with new bone regeneration [6, 7]. Furthermore, when combining the growth factors in scaffold, better recovering results are expected [8–13]. That will be very attractive for bone TB therapy in the future.

Ofloxacin has antitubercular activity and has lower hepatotoxicity than the traditional first-line antitubercular drugs (ATD). Moreover, comparing more and more TB involving strains resistant cases to the first-line ATD [14, 15], only a few cases reported the strain resistant to ofloxacin. That makes it currently the most commonly used agents against TB [16], especially for treating patients with underlying chronic liver diseases [17]. To encapsulate this drug effectively is important for the bone TB local drug release systems [18–20].

Until now, emulsification is still the most common method for drug encapsulation [21, 22]. However, the hydrophilic ofloxacin always results in the low drug loading efficiency in hydrophobic polymeric drug carriers such as polylactide-glycoside and also faces high burst release [18, 19]. Approaches used to prevent the hydrophilic drugs partitioning to the external aqueous phase during emulsification and hardening procedure include presaturation of external phase with drug, altering the aqueous phase pH and using more water-miscible solvents [23–27]. But the efficiency of all these endeavors is still needed to be improved.

Ofloxacin is positively charged drug at the pH value of body fluid. The negatively charged polyelectrolyte would embed ofloxacin in it. Migration of the macromolecular would be more difficult than the small drug molecular during the emulsification procedure. That is hypothesized to retain more ofloxacin in the final microsphere. This would be another alternative method to improve hydrophilic drug loading efficiency.

In this paper, ofloxacin was loaded in PLGA microparticles through water-in-oil-in-water emulsification solvent evaporation technique. We were going to add the HA internal phase additives to increase the drug retaining in the microparticles. Positively charged Pls was added and none additive microspheres were also fabricated to compare the loading efficiency and to testify the hypothesis. The effects of physical adsorption and electrostatic interaction of the polyelectrolytes with the drug on final loading efficiency and release behavior would be discussed.

## 2. Materials and Methods

### 2.1. Materials

PLGA (50/50, Mn = 60000) with an intrinsic viscosity of 0.78 dL g^−1^ was purchased from Jinan Daigang Biomaterial Co. Ltd. Hyaluronic acid (HA) was purchased from Shanghai Qisheng Biological Preparation Co. Ltd. Poly-l-lysine (Pls) was purchased from Sigma. Methyl cellulose (MC) was purchased from Sinopharm Chemical Reagent Co. Ltd. Dichloromethane (DCM) was purchased from Guangzhou Chemical Reagent Factory.

### 2.2. Preparation of Ofloxacin Loaded Microspheres

PLGA microspheres were prepared by a water-in-oil-in-water double emulsification solvent evaporation technique as the following steps. 500 *μ*L ofloxacin/PBS solution (10% w/v) was added into 5 mL PLGA/DCM (10%, w/v) solution and emulsified with the speed of 7200 rpm for 10 minutes using a high-speed stirrer (T10, IKA, German). Then, the primary emulsion was added into 20 mL MC solution (0.5%, w/v) and emulsified for another 10 minutes with the same speed. The emulsions were then transferred into 400 mL 35°C deionized water stirred for 4 h with the speed of 800 rpm and filtered to get the PLGA particles. Finally, The drug loading PLGA spheres were dried at 30°C for 24 hours.  Table 1 displays the formulation of the control group and the HA and poly-l-lysine microspheres with different internal phase amounts added in the ofloxacin/PBS solution.

### 2.3. Material Characterization

The sphere size distribution was measured using a laser diffraction particle size analyzer (Mastersizer 2000, Malvern, UK). The sample was dispersed ultrasonically in deionized water with stirring speed of 2000 rpm, adding samples until an obscuration rate of 5–18% was obtained. Background and sample were measured for 12 s. Optical properties of the sample were defined as follows: refractive index 1.460 and absorption 0.00. Each sample was measured in triplicate. Span index was calculated according to

(1) $$\begin{array}{c} \mathrm{span}\;\text{index}=\frac{D\left[v,90\right]-D\left[v,10\right]}{D\left[v,50\right]}. \end{array}$$

*D*[*v*, 90], *D*[*v*, 10], and *D*[*v*, 50] are the diameters at the particle accumulated volume ratio of 90%, 50%, and 10%, respectively (Table 2).

The microsphere surface morphology was examined by an environmental SEM (quanta 200, FEI, American). The samples were observed by simply being mounted onto double-sided adhesive tape on the sample stage and with gold presputtering. The test was performed in the low vacuum model (60 Pa) at the acceleration voltage of 10 Kv.

0.5% (Wt/V) sample solution was filled in the testing cell and measured by a potential meter (Malvern Nano ZS) at the temperature of 25°C under the applied voltage of 80 V. Each sample was measured for three times to get the mean value of zeta potential.

### 2.4. Loading Efficiency Determination

The calibration curve was prepared at the maximum ofloxacin absorption peak of 293.8 nm before the experiment by using an ultraviolet-visible spectrophotometer (Lambda950, Perkinelmer, USA). The equation regarding the calibration curve was *y* = 13.21847∗*x* − 0.55224 with the *R* ^2^ = 0.9997. In the equation, *y* refers to the absorbance data read from the spectrophotometer and *x* is the concentration of the prepared ofloxacin solution.

Ofloxacin loading efficiency of PLGA microsphere was measured as the following steps. 0.05 g sphere samples were dissolved into 5 mL DCM. 10 mL deionized water was added into the solution to extract the ofloxacin. Then the aqueous solution with the extracted drug was measured at the 293.8 nm to determine the loading amount according to the calibration curve. Drug incorporation efficiency was expressed as in (2). The individual values for three replicate determinations and their mean values were reported:

(2) Loading  Efficiency(%) =mass  of  drug  in  particlesmass  of  drug  used  in  formulation×100.

### 2.5. Drug Release Study

50 mg PLGA microsphere was suspended in 6 mL PBS solution. The microsphere suspensions were generally bathed in water at 37°C. At predetermined time intervals, samples were centrifuged at 2000 rpm for 5 min. 5 mL supernatant was taken for analyzing ofloxacin concentration. After this, 5 mL PBS was added in microsphere suspensions. All samples were taken in triplicate.

### 2.6. Particle Size and Distribution of PLGA Microsphere

**Figure 1:**
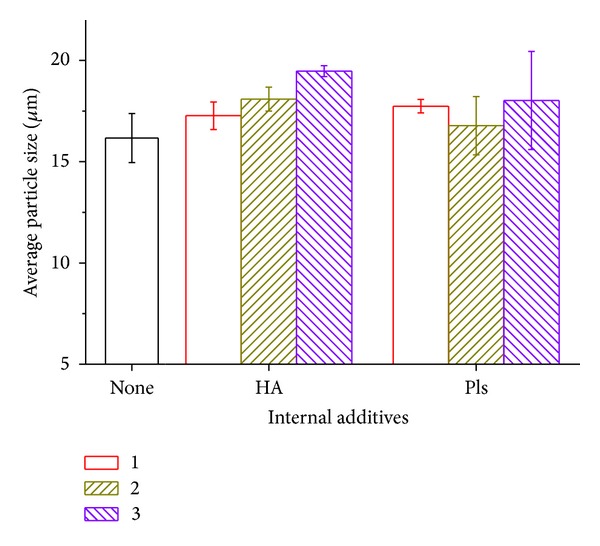
Average particle sizes of ofloxacin-loaded PLGA microspheres with varying amounts of internal additives.

Figure 1 displays the average particle size of the drug loaded PLGA microspheres without and with different varying amounts of internal polyelectrolytes. The mean particle sizes of different groups are in the range of 17 *μ*m~20 *μ*m. No big difference is noted among all of them, except the HA groups. The polyelectrolytes added in the internal aqueous phase would increase the aqueous phase osmatic pressure/viscosity and result in difficult aqueous dispersion and larger partial size. As that reported in the paper, the particle size depends largely on processing condition, including mixing shear force, solution mixing ratio, temperature, surfactant amount, and osmatic pressure [20]. In this paper, aqueous solution osmatic pressure of HA groups has increased, since the HA concentration in the aqueous solution resulted in relatively high osmatic pressure. It was also noticed that the solution viscosity of HA groups increased during the experiment.

**Figure 2:**
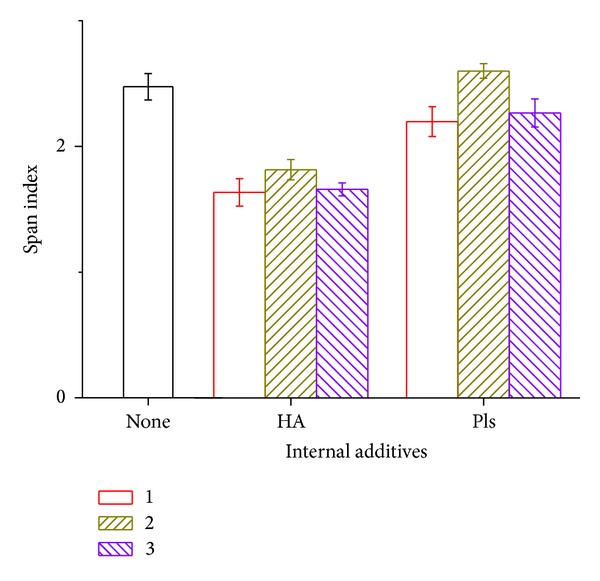
Span index of ofloxacin-loaded PLGA microsphere with varying amount of internal additives.

Figure 2 displays the particles span index. It reflects the particle size polydispersion according to (1). The particle distribution is affected by the internal phase additives. The span index of HA internal phase group is smaller than the control group since the HA solved inside the aqueous solution increased the viscosity and osmatic pressure. It suggested that the higher the internal phase viscosity, the more difficult for the emulsification procedure to get a wide-range microparticles. Since the viscosity of polylysine aqueous solution is not as high as HA aqueous solution, so the impact of polylysine on the particle span index is not as high as that of HA.

### 2.7. Morphological Examination

**Figure 3:**
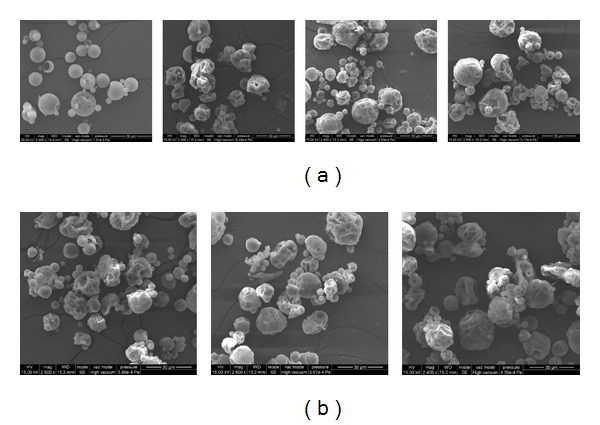
Surface morphology of ofloxacin-loaded microsphere ((a): none, HA-1, HA-2, and HA-3, (b): Pls-1, Pls-2, and Pls-3).

**Figure 4:**
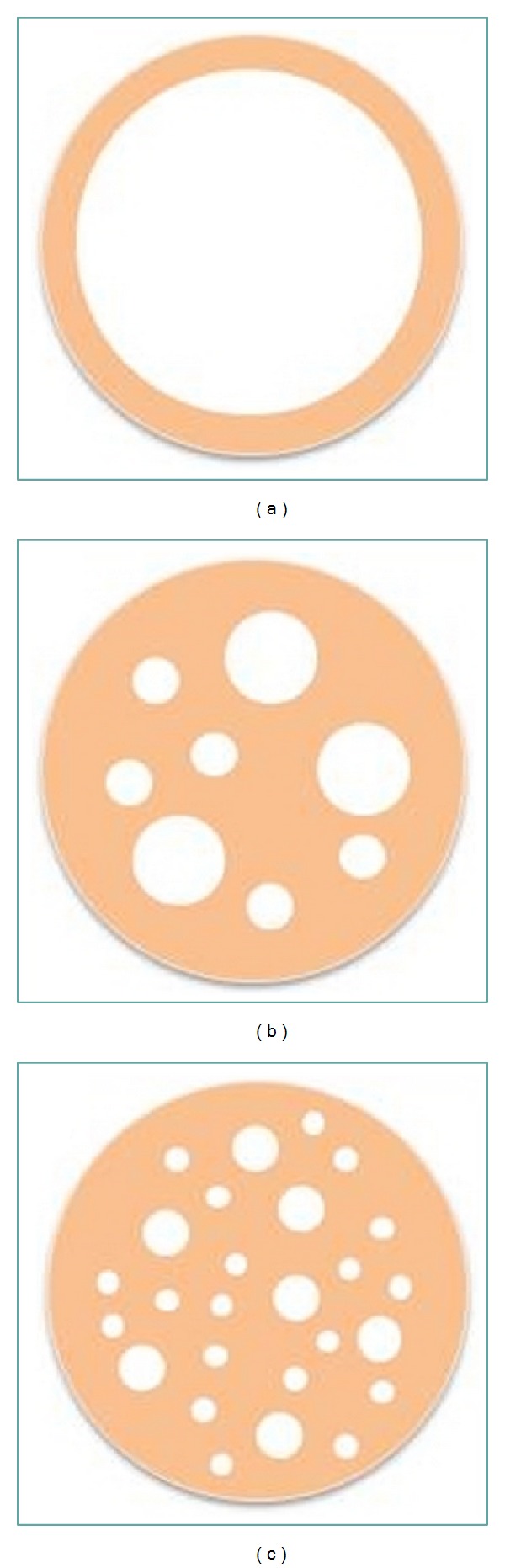
Sketch of the main type of internal morphology for microparticles.

Figure 3 displays the surface morphology of the microparticles produced by this double emulsion and evaporation method. The surface of PLGA microsphere is smooth and without visible pores when observed by the environment SEM without gold pre-sputtering (Figure 3(a): none). But after vacuum gold sputter coating, collapsed and wrinkled surface morphology could be observed as those displayed in Figure 3. This could be resulted by the microsphere internal cavities. The internal structure varied from dense solid to empty cavity, multivesicular structure, and matrix like structure depending on the primary emulsion stability [28, 29], as that displayed in Figure 4. With those internal cavities, when the vacuum chamber ventilated after gold coating, the interior and exterior pressure imbalance of the microparticles would cause the surface collapsing. The microcavities would provide more spaces for hydrophilic drug loading. But the uncontrollable internal structure would also result in uneven loading efficiency in different batches.

### 2.8. Loading Efficiency

**Figure 5:**
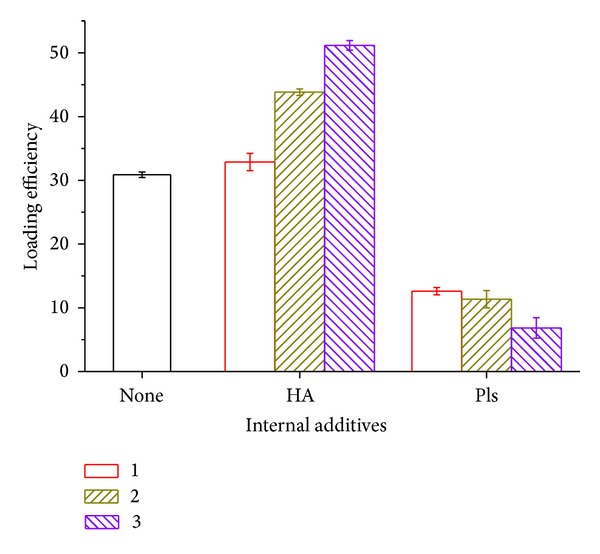
Loading efficiency of PLGA microsphere with varying amount of internal additives.

Figure 5 displays the encapsulation efficiency when HA and polylysine are added in the inner aqueous phase. Ofloxacin loading efficiency in HA internal phase group is higher than the control group and increased with its amounts inside, from 33% to 51%. This could be the reason of the interaction between the HA macromolecular and the drug. The zeta potential of the HA, polylysine, and the drug is 24 mV, 2.5 mV, and −14 mV, respectively, measured by a Malvern Nano sizer-potential meter.

HA is an acidic mucopolysaccharide. Negatively charged carboxyl groups on the HA would facilitate it to combine positively charged drugs on it through the electrostatic attraction. The entanglement of the HA molecular increases the internal phase viscosity and prevents the emigration of HA to the external aqueous solution during emulsification procedure. This helps to retain more drugs inside the internal phase.

When polylysine was added into the internal aqueous phase, the loading efficiency decreased dramatically as that in Figure 5. When the polylysine amount increased to 1%, ofloxacin encapsulation was decreased to 6.84% in PLGA microsphere. Besides the repulsion between the positively charged drug and the positively charged polylysine, the emulsification efficiency decreasing of the anionic surfactant MC in Pls groups could be another reason for this.

The emulsification efficiency is directly related to the macroscopical phase separation time. The macroscopical phase separation time is the time of an emulsion which becomes two phases when this could be observed by naked eye. This describes the stability of the primary emulsion. The average times of the initial macroscopic phase separation for none, HA, and Pls internal phase microsphere were 32 minutes, 67 minutes, and 12 minutes, respectively. The unstable primary emulsion of Pls group results in easier demulsification in the final microparticle formation procedure and lower drug loading efficiency. Furthermore, the electrostatic repulsion between polylysine and ofloxacin will speed the drug migration into the external aqueous solution following the demulsification.

### 2.9. In Vitro Release Study

**Figure 6:**
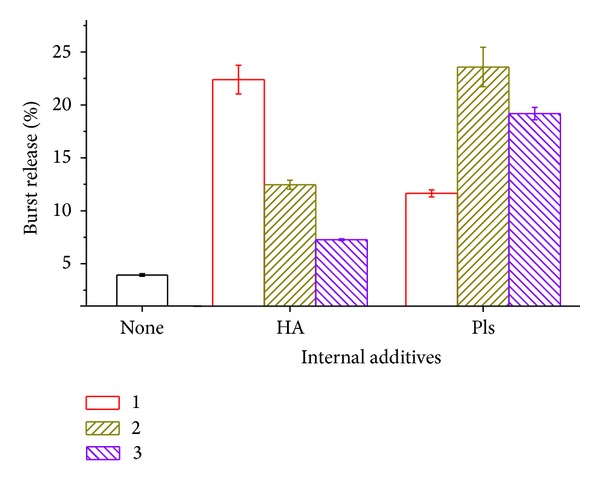
Burst release of PLGA microsphere with varying amount of internal additives at the first day.

Figure 6 presents the burst release of day 1. All groups with polyelectrolytes additives have the higher burst release than the control group. The burst release is mainly caused by the drug adhesive on the surface. During the microsphere fabrication procedure, the drug distributed in the external aqueous solution would be adsorbed onto the formed microspheres' surface. Furthermore, since the HA and polylysine were added in the formulation, the embedded polyelectrolytes in the microspheres would increase the surface hydrophilicity. This would result in more drug surface adsorbed on the particle.

Surface drug adsorption depends on the drug concentration in exterior aqueous solution. As we know from Figure 5, the lowest loading efficiency, the highest drug concentration in exterior aqueous solution. More drugs would be probably adsorbed on the microsphere surface. The drug adsorption and desorption are also influenced by the charged polyelectrolytes embedded in the microspheres.

Negatively charged HA would facilitate the adsorption and hindering the desorption. So, the higher HA inside the microsphere, the lower exterior drug concentration and the least burst release observed. In contrast, positively charged Pls would speed the desorption of the adsorbed drug. High burst release of the Pls groups was observed in the experiment.

**Figure 7:**
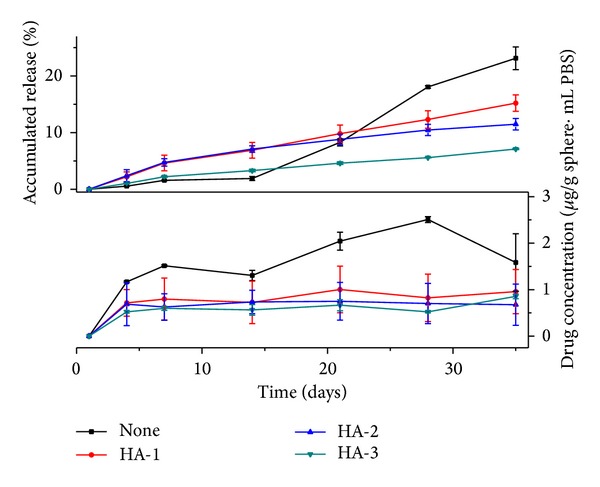
Release profile of ofloxacin/HA/PLGA microparticles.

Figure 7 displays the released profiles of the microparticle with different HA amounts in the internal phase for 35 days. With deduction of the burst release, the ofloxacin cumulated releases for 34 days were 15.2% of HA-1, 11.4% of HA-2, and 7.1% of HA-3, respectively. The cumulated release of the none additive microsphere was 23.1% at that time. The release speed of all the HA internal phase microspheres was slower than none additive microsphere. It was also noticed that the release speed decreased with the increase of HA amounts added into the internal phase.

Though the HA entrapped in the PLGA membrane material would improve particles' hydrophilicity and speed up PLGA degradation, a slower releasing is observed from Figure 7. That could be caused by the electrostatic attraction between the negative HA and the positive drugs. Furthermore, the slow migration of the HA from the PLGA entrapment is also retarding drug's diffusion from the capsules. The releasing speed could be controlled to be very slow as the HA contents increased to 0.7% in the internal phase by this method.

The minimum inhibitory concentration (MIC) of ofloxacin against the typical tuberculosis bacterium of mycobacterium is 1.0-2.0 *μ*g/mL [30]. Figure 7 displays that the drug concentration in the solution is about 1.0 *μ*g/g microsphere·mL. The drug concentration in the solution depends on two factors, the release rate and drug loading amounts in the microspheres. Though the release rate of the HA-3 internal phase group is slow, the loading amount is high; this group can still maintain the proper drug concentration in the solution.

When the microsphere is added in the tissue engineering scaffold or used directly as a microsphere scaffold, the total weight of the microsphere should be properly adjusted regarding the size of the implanting cavity.

**Figure 8:**
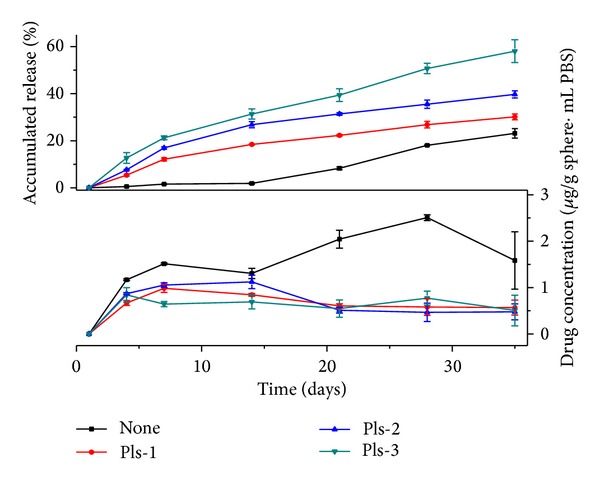
Release profile of ofloxacin/polylysine/PLGA microparticles.

Figure 8 presents the release pattern of the microparticles with different polylysine amounts in inner phase for 35 days. With deduction of the first-day burst release, the ofloxacin cumulated releases for 34 days are 30.1% of polylysine-1, 39.6% of polylysine-2, and 58.1% of polylysine-3, respectively. The release pattern is different as that of HA internal phase microsphere. The release speed of all polylysine internal phase microsphere is faster than none additive microsphere and the release speed increased with the increase of HA amounts added into the internal phase. The polylysine entrapped in the PLGA membrane material would improve particles' hydrophilicity and speed up PLGA degradation. In addition to that, the electrostatic repulsion between the positively charged polylysine and the positive drugs would benefit the drug diffusion to the external solution. Figure 8 displays that the drug concentration in the solution is about 1.0 *μ*g/g microsphere·mL. The concentration falls in the MIC range. The cumulated releasing is high in this group. The total sustain releasing time is shorter than the HA internal phase microspheres.

## 3. Discussion

Biodegradable polylactide and polylactide-co-glycolide polymers have been widely and intensively investigated for the control release of many drugs. However, the low entrapment efficiency of hydrophilic drug by these hydrophobic polymers is still a big problem since those drugs in general have very low affinity to them. Those would result in the drugs moving from the particles to the outer aqueous phase when they were produced by emulsion methods.

Several works had been done in improving this hydrophilic drug's loading efficiency in PLGA microsphere, such as altering the emulsion stabilizer, and changing emulsification time, changing the dilution volume between aqueous and organic phase [29, 31, 32]. The most efficient method to solve this problem is to increase PLGA concentration in DCM solution. But the preparation process was hard to control since that the higher PLGA concentration in the solution would result the higher solution viscosity. And at the same time, many visible pores in the microsphere surface result in as high as 45% burst release [29].

In the future, multipurpose sustained release scaffold implanted into the cavity after surgical bone infection foci debridement should be a promising expectation for bone tuberculosis therapy. This complicated multipurpose carrier system might probably involve hydrophilic, hydrophobic, positively and negatively charged drugs, and bioactive factors. Their sustained and/or procedure releasing would be a big challenge.

In this paper, we presented the possibility that the loading efficiency and release profile could be adjusted by changing the internal phase. This provides alternatives for future sustained hydrophilic drug and protein control release besides previous methods. The method described in the paper also makes it easier for us to tailor the drug releasing profile together with the other methods to gain the best therapy effect.

## 4. Conclusions

We have demonstrated effects of internal phase polyelectrolytes on the encapsulation efficiency, initial burst, and release profile of the hydrophilic drug ofloxacin. Promoting hydrophilic drug loading efficiency was dominated by adsorption and electrostatic attraction between the internal phase additives and the drug. Release profile and initial burst release were a balance between the wall materials' degradation and the interactions between the internal phase additives and the drug. As for the bone tuberculosis therapy, the 0.7% HA internal phase presented the lowest initial releasing, longest releasing time, and relatively high loading efficiency. That could be a promising regiment for that disease therapy.

The results of this research also revealed the importance of the internal phase controlling of the microparticles. When combining the controlling wall material's degradation with this method, that will be easier to fabricate a multipurpose in situ drug releasing scaffold for clinic application in the future.

## Figures and Tables

**Table 1:** Concentration of internal phase additives.

Internal phase	None (Wt/V%)	Group 1 (Wt/V%)	Group 2 (Wt/V%)	Group 3 (Wt/V%)
Hyaluronic acid	0	0.1	0.3	0.7
Polylysine	0	0.25	0.75	1.0

**Table 2:** Diameters at the particle cumulated volume ratio of 90%, 50%, and 10%.

	*D*[*v*, 10] (*μ*m)	*D*[*v*, 50] (*μ*m)	*D*[*v*, 90] (*μ*m)
None	5.24 ± 0.43	16.17 ± 1.21	45.18 ± 1.08
HA-1	6.82 ± 0.67	17.28 ± 0.68	35.13 ± 3.16
HA-2	6.75 ± 0.35	18.09 ± 0.59	39.57 ± 0.65
HA-3	7.75 ± 0.43	19.47 ± 0.27	40.06 ± 0.09
Pls-1	8.29 ± 0.81	17.74 ± 0.33	47.29 ± 1.01
Pls-2	6.90 ± 0.57	16.78 ± 1.44	50.55 ± 0.38
Pls-3	8.55 ± 0.82	18.02 ± 2.42	49.46 ± 0.67
